# Susceptibility to Ventricular Arrhythmias Resulting from Mutations in *FKBP1B*, *PXDNL*, and *SCN9A* Evaluated in hiPSC Cardiomyocytes

**DOI:** 10.1155/2020/8842398

**Published:** 2020-09-01

**Authors:** Hector Barajas-Martinez, Maya Smith, Dan Hu, Robert J. Goodrow, Colleen Puleo, Can Hasdemir, Charles Antzelevitch, Ryan Pfeiffer, Jacqueline A. Treat, Jonathan M. Cordeiro

**Affiliations:** ^1^Department of Experimental Cardiology, Masonic Medical Research Institute, Utica, NY, USA; ^2^Department of Cardiovascular Research, Lakenau Institute for Medical Research, Wynnewood, PA, USA; ^3^Department of Cardiology & Cardiovascular Research Institute, Renmin Hospital of Wuhan University, Wuhan, China; ^4^Department of Cardiology, Ege University School of Medicine, Izmir, Turkey; ^5^Kimmel College of Medicine of Thomas Jefferson University, Philadelphia, PA, USA

## Abstract

**Background:**

We report an inherited cardiac arrhythmia syndrome consisting of Brugada and Early Repolarization Syndrome associated with variants in *SCN9A*, *PXDNL*, and *FKBP1B*. The proband inherited the 3 mutations and exhibited palpitations and arrhythmia-mediated syncope, whereas the parents and sister, who carried one or two of the mutations, were asymptomatic.

**Methods and Results:**

We assessed the functional impact of these mutations in induced pluripotent stem cell cardiomyocytes (hiPSC-CMs) derived from the proband and an unaffected family member. Current and voltage clamp recordings, as well as confocal microscopy analysis of Ca^2+^ transients, were evaluated in hiPSC-CMs from the proband and compared these results with hiPSC-CMs from undiseased controls. Genetic analysis using next-generation DNA sequencing revealed heterozygous mutations in *SCN9A*, *PXDNL*, and *FKBP1B* in the proband. The proband displayed right bundle branch block and exhibited episodes of syncope. The father carried a mutation in *FKBP1B*, whereas the mother and sister carried the *SCN9A* mutation. None of the 3 family members screened developed cardiac events. Action potential recordings from control hiPSC-CM showed spontaneous activity and a low upstroke velocity. In contrast, the hiPSC-CM from the proband showed irregular spontaneous activity. Confocal microscopy of the hiPSC-CM of the proband revealed low fluorescence intensity Ca^2+^ transients that were episodic in nature. Patch-clamp measurements in hiPSC-CM showed no difference in *I*_Na_ but reduced *I*_Ca_ in the proband compared with control. Coexpression of *PXDNL*-R391Q with *SCN5A*-WT displayed lower *I*_Na_ density compared to *PXDNL*-WT. In addition, coexpression of *PXDNL*-R391Q with *KCND3*-WT displayed significantly higher *I*_to_ density compared to *PXDNL*-WT.

**Conclusion:**

*SCN9A*, *PXDNL*, and *FKBP1B* variants appeared to alter spontaneous activity in hiPSC-CM. Only the proband carrying all 3 mutations displayed the ERS/BrS phenotype, whereas one nor two mutations alone did not produce the clinical phenotype. Our results suggest a polygenic cause of the BrS/ERS arrhythmic phenotype due to mutations in these three gene variants caused a very significant loss of function of *I*_Na_ and *I*_Ca_ and gain of function of *I*_to_.

## 1. Introduction

Cardiac myocytes derived from human-induced pluripotent stem cells (hiPSC-CM) can be used for a variety of applications such as regenerative therapy, cardiac safety pharmacology, and as models of human genetic disease. Specifically, patient-derived *in vitro* models can be used to study diseases of cardiac ion channels to determine how mutations in these channels alter the cardiac action potential [1]. Using this technology, hiPSC-CM can be generated in large quantities to elucidate the functional alterations resulting from the mutations. However, the disadvantage is that hiPSC-CM are immature, both electrophysiologically and morphologically [2, 3], suggesting that results obtained in hiPSC-CM may not translate to the adult phenotype of the disease.

It is well established that the development of cardiac arrhythmias is linked to mutations in genes that encode cardiac ion channels. These arrhythmias occur in both atria and ventricles and can appear in the absence of any structural defect. Long QT, Short QT, and Brugada Syndrome are all conditions linked to ion channelopathies [4]. For example, Short QT is characterized by QT intervals less than 330 ms, and 8 different genes have been linked to Short QT syndrome [5]. Brugada Syndrome (BrS) is characterized by right bundle branch block and ST segment elevation in the right precordial leads (V1-V3) of an electrocardiogram [6], and 21 genes have been associated with BrS [7]. These inherited arrhythmia syndromes demonstrate that the majority of these genes encode cardiac ion channels resulting in a clear structure-function alteration in the channel protein. In addition, other genes encode auxiliary subunits which do not conduct ions but are known to associate with ion channels and are important for channel gating and trafficking [8].

The manifestation of many arrhythmia syndromes is due to an imbalance in depolarizing and/or repolarizing currents during the cardiac action potential (AP). These imbalances result in a decrease in inward *I*_Na_ or *I*_Ca_ or an increase in outward *I*_K_ which would affect the morphology of the action potential resulting in the various syndromes [9, 10]. Recent studies have shown that mutations in genes not thought to be associated with cardiac ion channels can precipitate cardiac arrhythmias. For example, it is well documented that mutations in Ankyrin B are responsible for certain Long QT phenotypes [11, 12]. Moreover, it is clear that multiple genes may be involved in the development of cardiac arrhythmias highlighting the polygenic nature of these diseases [13, 14].

In this study, we identified a family that carried several mutations in genes not thought to be related to cardiac ion channels. One of these mutations was in the voltage-gated sodium channel alpha subunit 9 (*SCN9A*), a gene that encodes the neuronal sodium channel Nav1.7. A second mutation was found in a gene that encodes a peroxidasin-like protein (*PXDNL*). The third mutation was found in FK506-binding protein 1B (*FKBP1B*). FK506 is a commonly used immunosuppressant that binds to FK506-binding protein, and FKBP1B plays a role in heart failure and genetic forms of arrhythmias [15]. Interestingly, only the proband who inherited the 3 damaging mutations showed characteristics of Early Repolarization Syndrome (ERS), BrS, and right bundle branch block (RBBB) as well as numerous cardiac arrhythmias. We utilized hiPSC cardiomyocytes derived from the proband and an unaffected family member to systematically study the functional effects of these 3 mutations.

## 2. Methods

### 2.1. Subjects

IRB approval and informed consent were obtained from the family included in the study. The proband was identified following a visit to a physician due to episodes of palpitations and syncope, and ECG analysis initially revealed an ERS ECG. We studied three additional members of his family by genetic analysis: his sister and his parents.

### 2.2. Next-Generation Sequencing

An Ion Torrent Personal Genome Machine (PGM) was utilized to perform high-throughput sequencing (HTS) which gave us the capability to target and sequence 87 candidate genes at a time responsible for Brugada Syndrome [6, 8], Long QT [16], Short QT Syndrome [17, 18], and Early Repolarization Syndrome [10, 19, 20]. These candidate genes encode ion channels and other proteins including pumps, exchangers, calcium-handling proteins, gap junctional proteins, and structural proteins. They were selected based on their relative expression in human neuronal and cardiac tissue and their functional roles in generating action potentials and altering excitability.

The Ion Torrent PGM (Life Technologies, Carlsbad, CA) sequenced genomic DNA fragments generated through the use of Custom Ion Ampliseq 2.0 (Life Technologies, Carlsbad, CA). The Coding Exons as well as intron borders for our 87 genes of interest from human genomic DNA were amplified through a massive multiplex PCR approach. DNA libraries were prepared by attaching adapters to these fragments, as well as a unique molecular barcode for each sample. Library quality was assessed using the Qubit 2.0 Fluorometer and the dsDNA HS Assay (Life Technologies, Carlsbad, CA). An Ion Chef automation system then performed emulsion PCR, enrichment, and chip loading, followed by direct sequencing with the Ion Torrent PGM.

### 2.3. Mutation Confirmation

All rare variations and mutations uncovered were confirmed using gold standard Sanger sequencing. PCR products were purified with a commercial enzyme (ExoSAP-IT, USB, Cleveland, OH) and directly sequenced from both directions using Big Dye Terminator 3.1 chemistry on an Applied Biosystems 3730 DNA Analyzer (Life Technologies, Carlsbad, CA).

### 2.4. Bioinformatics Analysis

After signal processing and basecalling, the Ion Torrent Suite software was used to map the sequencing reads to the DNA reference sequence [hg19] and identify variants through the VariantCaller plugin as well as the IonReporter analysis tool. Run Quality was assessed using the Coverage Analysis plugin which reports average depth of coverage, uniformity of coverage, and percent on target as well as other metrics. IonReporter compares all variations identified against NCBI's dbSNP to rule out common SNP's, as well as the 1000 genomes project and Exome Sequencing Project (ESP) to get published frequencies. Variants of interest were then flagged for verification through Sanger sequencing. Mutations and rare variants are analyzed using several pathogenicity prediction tools such as PolyPhen2, SIFT, and Grantham. When available, family members are sequenced for these mutations and rare variants to analyze the penetrance and establish a genotype-phenotype correlation.

### 2.5. Generation of hiPSC-CM

The control and patient-derived human iPS cells were reprogrammed from fibroblasts with Oct4, Sox2, Lin28, and Nanog. A directed differentiation protocol to derive cardiomyocytes using serum-free, chemically defined media supplemented with CHIR99021, IWP2, Activin A, and KY0211 in stage specific manner, as previously described [21]. This protocol yielded contractile clusters by days 9 to 12 postdifferentiation. Monolayers ranging between 20 and 50 days of maturity were plated on matrigel-coated dishes and maintained with RPMI B27+ until use.

### 2.6. Fluorescence Imaging

Fluo 4-AM was used for this study. Fluo 4-AM dissolved in 20% F-127 pluronic in dimethyl sulfoxide (DMSO) for 20 min at room temperature as previously described [22, 23]. Confocal experiments were performed with an Olympus Fluoview laser-scanning confocal microscope. Fluo 4-loaded iPSC-CMs were placed in a perfusion chamber and excited at 488 nm using an argon laser, and fluorescence emission was detected via a 520 nm band-pass filter and photomultiplier tube. Confocal images were acquired with the Fluoview acquisition software program, and spontaneous activity was recorded on a personal computer for later analysis. Images acquired with Fluoview acquisition software were analyzed with ImageJ and Fluoview analysis software.

### 2.7. Cell Transfection/Mutagenesis

Site-directed mutagenesis was performed using QuikChange (Stratagene, LaJolla, CA) on full-length human wild type (WT) PXDNL cDNA cloned in pcDNA3. TSA201 cells were grown in DMEM with Glutamax supplemented with 10% FBS in 35 mm culture dishes and placed in a 5% CO_2_ incubator at 37°C. To determine whether the R391Q mutation in PXDNL altered the biophysical characteristics of *I*_Na_ or *I*_to_, TSA201 cells were cotransfected with a combination of mutant or WT PXDNL. To assess the effect on *I*_Na_, cells were cotransfected using FuGene6 (Roche Diagnostics, Indianapolis, IN) with a 1 : 1 molar ratio of WT human SCN5A, and WT or R391Q mutant PXDNL [24]. To assess the effect on *I*_to_, cells were cotransfected using FuGene6 (Roche Diagnostics, Indianapolis, IN) with a 1 : 1 molar ratio of WT human KCND3, and WT or R391Q mutant PXDNL. In addition, 0.20 *μ*g of enhanced green fluorescent protein cDNA was added to the transfection mixture. Cells displaying fluorescence 48–72 h after transfection were used for electrophysiological study.

### 2.8. Electrophysiology

Sharp microelectrodes (40–60 M*Ω*) filled with 2.7 M KCl were used to record APs from spontaneously beating monolayers, as previously described [25]. Monolayers were superfused with HEPES-Tyrode's solution of the following composition (in mM): NaCl 140, KCl 4, MgCl_2_ 1, HEPES 10, D-Glucose 10, and CaCl_2_ 2; pH was adjusted to 7.4 with NaOH. The microelectrodes were connected to an MultiClamp 700B amplifier (Molecular Devices, Foster City, CA, USA) operating in current clamp mode. All signals were digitized (sampling rate = 50 kHz), stored on a computer, and analyzed using pClamp9 acquisition suite (Molecular Devices, Foster City, CA).

Voltage clamp recordings were made using patch pipettes fabricated from borosilicate glass capillaries (1.5 mm O.D., Fisher Scientific, Pittsburg, PA). The pipettes were pulled using a gravity puller (Model PP-830, Narashige Corp.) and filled with pipette solution of the following composition (mM): for *I*_Ca_ recordings, the patch pipette contained (in mM) CsCl 120, MgCl_2_ 1.0, EGTA 10, MgATP 5, HEPES, and CaCl2 5, pH = 7.2 with CsOH [26]. For *I*_Na_ recordings in hiPSC-CMs, the external solution contained (in mM) N Methyl D-Glucamine 105, NaCl 40, CaCl_2_ 2.0, MgCl_2_ 1.0, glucose 10, HEPES free acid 10, CdCl_2_ 0.3, pH adjusted to 7.4 with NaOH/HCl. For *I*_Na_ recordings in transfected TSA cells, the perfusion solution contained (in mM) NaCl 130, KCl 5, CaCl_2_ 1.8, MgCl_2_ 1.0, sodium acetate 2.8, HEPES 10, glucose 10, pH 7.4 with NaOH. The pipette solution contained (in mM) NaCl 15, CsF 120, MgCl_2_ 1, KCl 5, HEPES 10, Na_2_ATP 4, and EGTA 10, pH adjusted to 7.2 with CsOH [25, 27]. For hiPSC-CM recordings, peak *I*_Na_ was dramatically reduced in the low extracellular Na^+^ solution to ensure adequate voltage control, as gauged by the slope of a Boltzmann fit to the steady-state activation curve [28]. Current signals were recorded using a MultiClamp 700A amplifier (Axon Instruments Inc., Foster City, CA), and series resistance errors were reduced by about 60-70% with electronic compensation. Signals were acquired at 20-50 kHz (Digidata 1322, Axon Instruments) and analyzed with a microcomputer running pClamp 9 software (Axon Instruments, Foster City, CA). All recordings were made at 37° C with the exception of I_Na_ which was performed at room temperature.

### 2.9. Statistics and Data Analysis

Ca^2+^ transient and electrophysiological data are presented as Mean ± SEM, and statistical comparisons were made using ANOVA followed by a Student-Newman-Kuels, or Student's *t*-test, as appropriate. Significance was determined at *p* < 0.05.

## 3. Results

**Figure 1:**
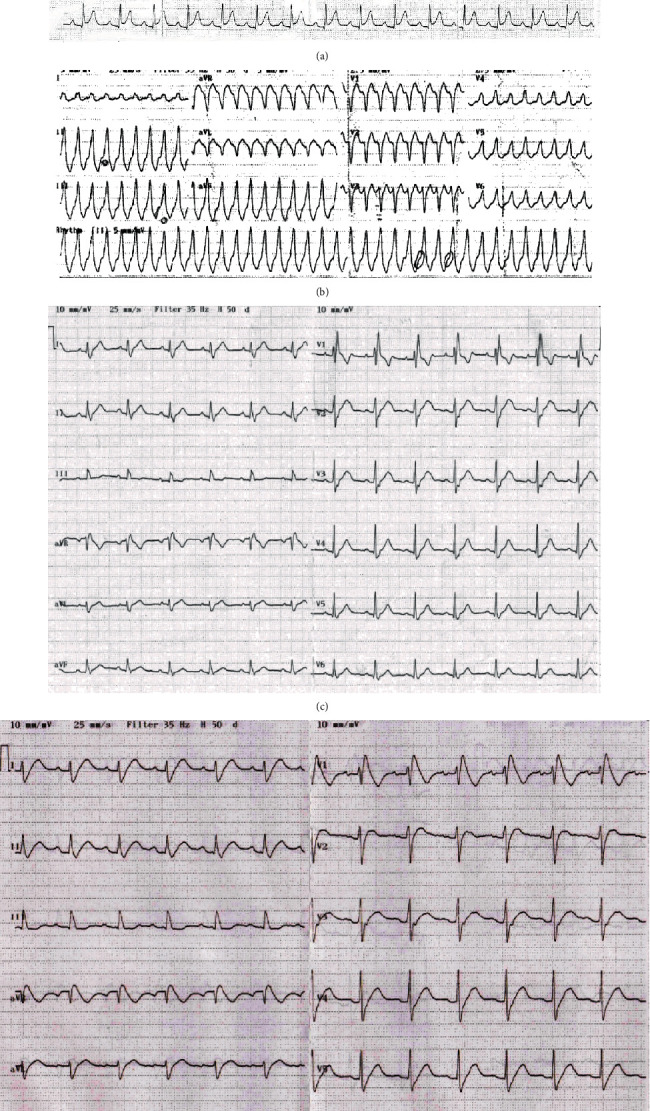
(a) ECG analysis of patient MMRL 1126 showing early repolarization phenotype. A prominent ST segment elevation and slurring of the QRS complex is apparent in several leads. (b) Infusion of dobutamine caused the appearance of monomorphic VT in MMRL1126 which persisted after dobutamine was discontinued. (c) The arrhythmogenic focus was ablated and patient subsequently exhibited RBBB apparent in lead V1. (d) Patient was challenged with ajmaline resulting in unmasking of BrS.

The proband (MMRL1126) was a male who initially presented with palpitations and syncope. ECG analysis of the patient revealed a slurring at the end of the QRS complex and ST-segment elevation leading to an initial diagnosis of ERS (Figure 1(a)). The patient was given dobutamine which resulted in sustained monomorphic VT and syncope originating from septal RVOT which persisted after dobutamine infusion was discontinued (Figure 1(b)). RBBBc was developed in the proband after radiofrequency catheter ablation in septal wall of RVOT (Figure 1(c)). Patient was challenged with ajmaline resulting in unmasking of BrS ECG phenotype (Figure 1(d)).

**Figure 2:**
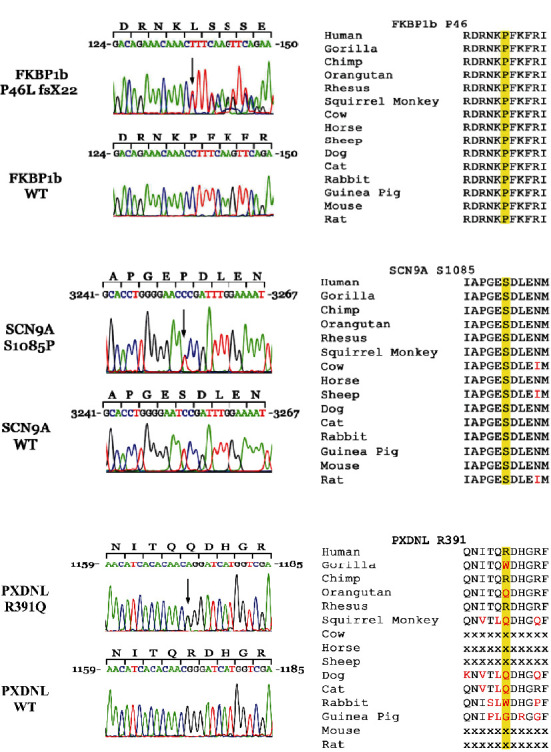
DNA sequencing analysis of SCN9A and FKBP1b. The *FKBP1b* gene was mutated to a C deletion at location 137 in exon 3 resulting in complexed amino acid changes. The amino acid change was proline to leucine at position 46; the next 22 amino acids were framed shifted culminating in a stop codon 22 amino acides downstream (P46L fsx22). The *SCN9A* gene was mutated from a T to C substitution at position 3253 in exon 17, resulting in an amino acid change from serine to proline at position 1085 (S1085P). The *PXDNL* gene was mutated from a G to A at position 1172 in exon 14, resulting in an amino acid change from arginine to glutamine at position 391 (R391Q). Mutations in *SCN9A* and *FKBP1b* are highly conserved across the various species. *PXDNL* is only expressed in certain mammals including primates.

**Figure 3:**
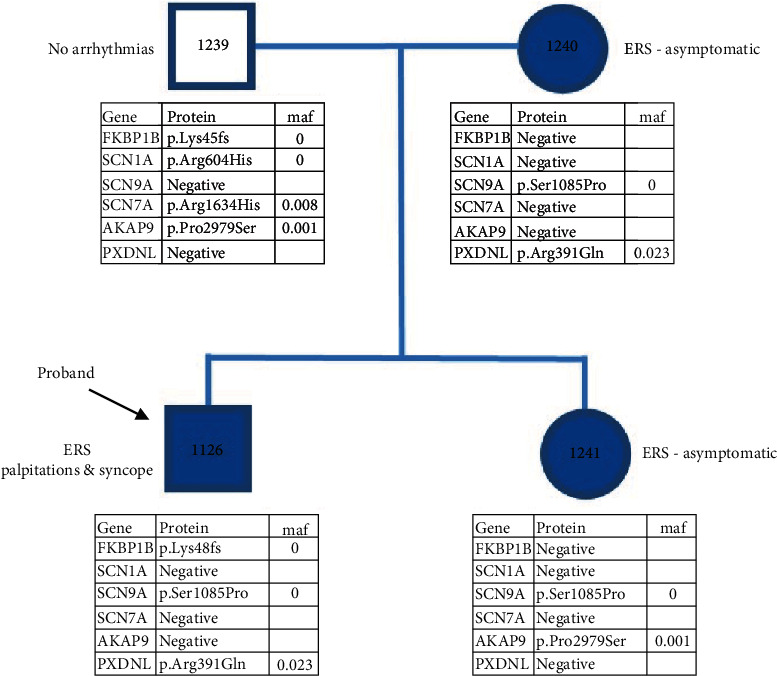
Pedigree of the proband (denoted with arrow) and family. Genetic analysis revealed that each parent carried different heterozygous mutation(s). The father (1239) carried the FKBP1b mutation, whereas the mother (1240) carried the PXDNL and SCN9A mutations. The sister of the proband (1241) only inherited the mutation in SCN9A. Both parents and the sister were asymptomatic.

We performed NextGen sequencing of the index patient as well as the sister and the parents. Genetic analysis of the index patient revealed three heterozygous exonic mutations, one in *SCN9A*, another in *PXDNL*, and the third in the *FKBP1b* gene (Figure 2). The *SCN9A* gene was mutated from a T to C substitution at position 3253 in exon 17, resulting in an amino acid change from serine to proline at position 1085 (S1085P). The *FKBP1b* gene was mutated to a C deletion at location 137 in exon 3 resulting in complexed amino acid changes. The amino acid change was proline to leucine at position 46; the next 22 amino acids were frame shifted culminating in a stop codon 22 amino acids downstream (P46L fsx22). The *PXDNL* gene was mutated from a G to A at position 1172 in exon 14 resulting in an amino acid change from an arginine to a glutamine at position 391 (R391Q). Mutations and polymorphisms of the proband are shown in Table 1 and Figure 3.

Pedigree analysis of the family showed that the mother of the proband carried both the *SCN9A* and *PXDNL* mutations, whereas the father carried the *FKBP1b* gene mutation, and both were found to be asymptomatic. The sister inherited only the *SCN9A* gene mutation and was also asymptomatic (Figure 3).

**Figure 4:**
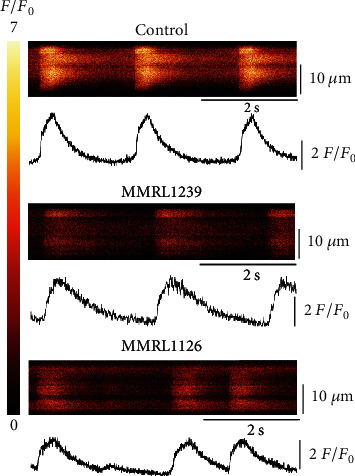
Line scans recorded from WT, MMRL 1239, and MMRL1126 hiPSC-CMs showing spontaneous Ca^2+^ transients in all hiPSC-CMs. However, fluorescence intensity was much lower in MMRL 1126, and spontaneous activity was episodic in nature.

Patient MMRL 1126 and unaffected family member MMRL 1239 carried a mutation in *FKBP1b* gene. The *FKBP1b* gene encodes for a binding protein, calstabin 2, which is closely associated with the cardiac ryanodine receptor (RYR2) and is believed to play a role in the regulation of RYR2 open probability. Mutations in the *FKBP1b* gene may affect Ca^2+^ release from the SR resulting in alterations in the Ca^2+^ transient as well as indirectly affecting action potential waveform and duration. As an initial basis of comparison, we recorded calcium transients in normal (WT) hiPSC-CMs and hiPSC-CMs derived from affected patient MMRL1126 and unaffected family member MMRL 1239 (see Figure 3). Cells were loaded with fluo-4 AM, and Ca^2+^ transients from spontaneously beating cells were recorded (Figure 4(a)). Compared to control and MMRL 1239 monolayers, Ca^2+^ transients from patient 1126 showed slower spontaneous activity and had a lower fluorescence intensity (Table 2). In addition, episodic or intermittent spontaneous activity was noted in a number of recordings (34/93). The lower fluorescence intensity suggests that there is less intracellular calcium present in MMRL1126 cells during the course of an action potential. Interestingly, even though MMRL 1239 carried the mutation in *FKBP1b*, the Ca^2+^ showed different characteristics compared to MMRL 1126 and seemed to resemble WT hiPSC-CM although some episodic transients were observed.

**Figure 5:**
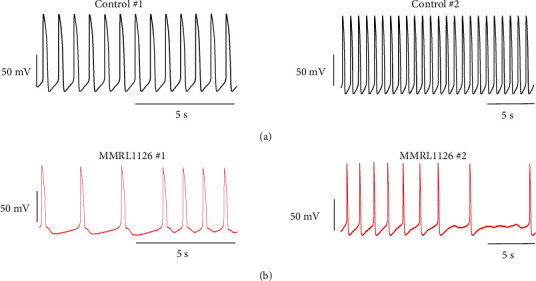
Action potential (AP) recordings obtained from WT and MMRL1126 monolayers. WT hiPSC-CMs were more hyperpolarized and had a larger *V*_max_. In addition, hiPSC-CMs from MMRL1127 exhibited slower spontaneous activity, and some showed episodic activity.

Since MMRL 1126 showed clinical symptoms, and hiPSC-CMs derived from this patient exhibited abnormal spontaneous activity, we systematically evaluated the mechanism for these differences. We further evaluated hiPSC-CM derived from MMRL 1126 by recording action potentials from WT and MMRL1126 hiPSC-CMs. Cells were plated at high density to form monolayers, and a detailed analysis of their electrophysiological characteristics was performed. Action potentials recorded from control monolayers were spontaneously active, exhibited a slow upstroke velocity, and no phase 1 repolarization. Recordings from hiPSC-CMs derived from the proband also showed spontaneous activity (Figure 5). However, the spontaneous activity was episodic, and brief periods of no spontaneous activity was also observed (8/13 monolayers). In monolayers derived from the proband, MDP was more depolarized (−57.8 ± 2.1 vs. −68.7 ± 1.1 mV; *p* < 0.05), and *V*_max_ was lower (17.3 ± 1.6 vs. 38.5 ± 2.4 V/s; *p* < 0.05) compared to controls. In addition, APD_50_ and APD_90_ were briefer than that observed in WT hiPCS-CMs (Table 3).

**Figure 6:**
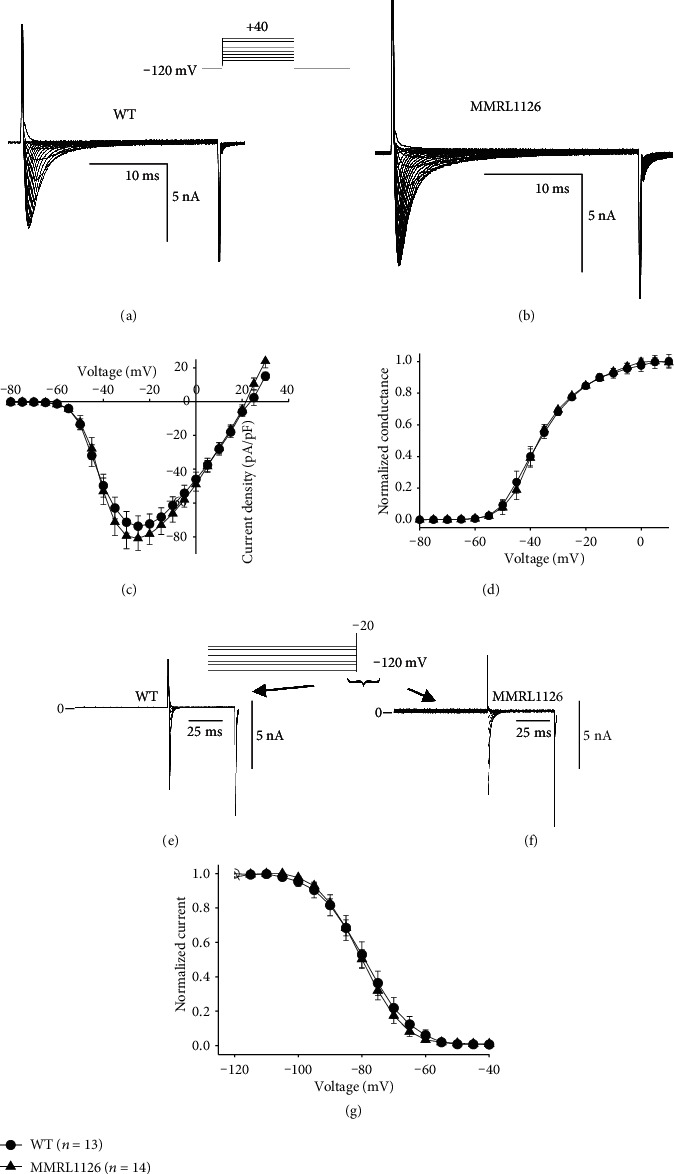
Representative *I*_Na_ recordings from a WT (a) and MMRL1126 hiPSC-CM (b). Current recordings were obtained at test potentials between −80 and 25 mV in 5 mV increments. The holding potential was −120 mV. (c) I–V relation for WT (*n* = 13) and MMRL1126 (*n* = 14) showing no difference in *I*_Na_ magnitude. (d) Steady-state activation relation for WT and MMRL1126 hiPSC-CM cells. Chord conductance was determined using the ratio of current to the electromotive potential for the cells shown in (a) and (b). Data were normalized and plotted against their test potential. Representative steady-state inactivation recordings from a WT (e) and MMRL1126 hiPSC-CM (f). The voltage clamp protocol is shown at the top of the figure. Peak current was normalized to their respective maximum values and plotted against the conditioning potential. The mean data for the steady-state inactivation relation is shown (g).

Genetic analysis revealed that the proband carried 3 mutations, one of which was in *PXDNL*. The *PXDNL* gene encodes for a peroxidasin-like protein which is expressed in the heart. Previous immunocytochemical studies of PXDNL protein demonstrated that the protein product is highly expressed on the membrane of human ventricular cells, especially at the intercalated discs [29]. In prior studies from our labs, we noted a similar expression pattern of Na^+^ channels in ventricular cells [30] suggesting that the PXDNL protein may colocalize with Na^+^ channels. This potential interaction may result in an alteration in the size of the Na^+^ current, contributing to the lower *V*_max_ measured in the hiPSC-CMs of the proband (Table 3). We next measured peak *I*_Na_ in WT and MMRL1126 hiPSC-CMs. Representative traces are shown in Figures 6(a) and 6(b). *I*_Na_ was activated by a series of depolarizing pulses from -80 to +30 mV in 5 mV increments. Analysis of the current-voltage relation showed that peak *I*_Na_ was −72.5 ± 6.5 pA/pF for WT and −80.9 ± 7.0 for MMRL1126 at -35 mV (Figure 6(c), *p* = N.S.). Steady state activation was not significantly different between WT and MMRL1126 (Figure 6(d)). Although *I*_Na_ current density was not significantly different between WT and MMRL1126, the lower *V*_max_ observed in hiPSC-CMs from MMRL 1126 could possibly be due to a shift in Na^+^ channel availability. We next evaluated steady-state inactivation between the two cell types. Peak current after a 500 ms prepulse was normalized to the maximum current and plotted as a function of the prepulse voltage. A Boltzman function was then fit to the data. Figures 6(e) and 6(f) shows representative traces recorded from a WT and MMRL1126 hiPSC-CM. The midinactivation potential was −79.3 ± 0.12 mV for WT and −80.0 ± 0.16 for MMRL1126 (*p* = N.S.) These results demonstrate that the difference in *V*_max_ between WT and MMRL1126 was not due to a shift in steady-state inactivation (Figure 6(g)) and suggest that the difference in *V*_max_ between WT and MMRL1126 is likely due to the more depolarized MDP in the proband.

**Figure 7:**
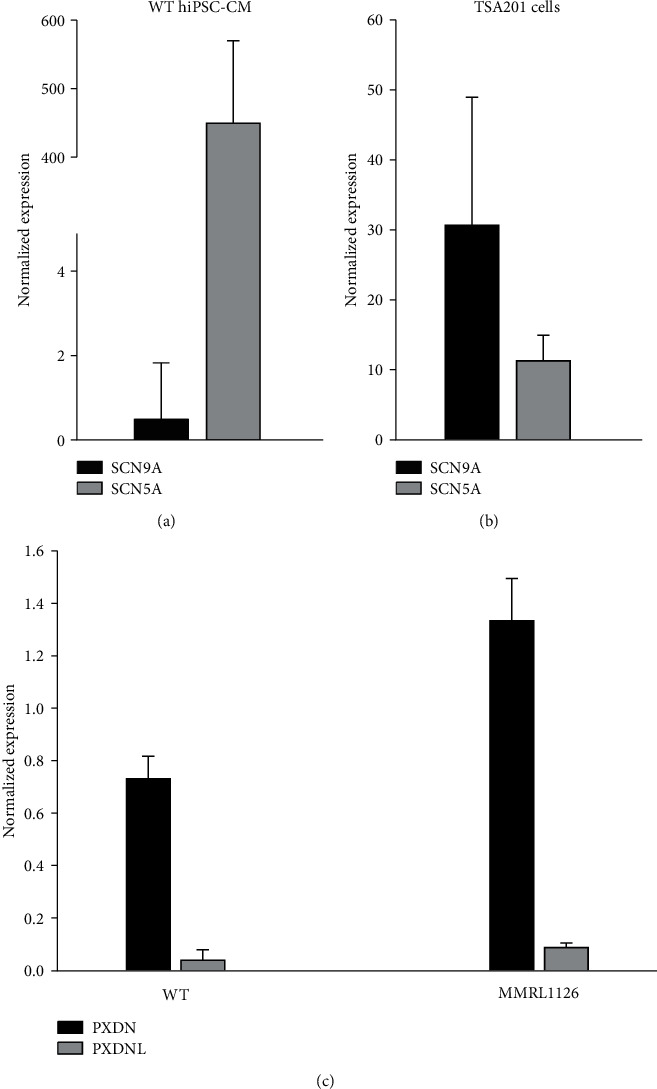
RT-qPCR analysis of SCN9A and SCN5A in hiPSC-CMs (normalized to GAPDH). SCN5A message was highly expressed, whereas levels of SCN9A were negligible in the hiPSC-CMs (a). RT-qPCR analysis of SCN9A and SCN5A in TSA201 cells transfected with SCN5A and SCN9A plasmid at a 1 : 1 molar ratio. SCN9A message could readily be detected in transfected TSA201 cells (b). RT-qPCR analysis of PXDN and PXDNL in hiPSC-CMs (c). PXDNL was expressed at low levels in both WT and MMRL1126 hiPSC-CMs.

The *SCN9A* gene encodes Nav1.7 sodium channels which are found mainly in nerve cells and transmit pain signals. Interestingly, most reported mutations in Nav1.7 result in either insensitivity to pain (due to a loss of function in this channel) [31] or a hypersensitivity to pain (due to a gain of function in this channel) [32]. Previous studies have shown the presence and functional role for neuronal or “brain type” Na^+^ channels in the heart although Nav1.7 was not probed in those studies [30, 33, 34]. We first used WT hiPSC-CMs and probed for both *SCN5A* and *SCN9A* to determine the level of transcript. RT-PCR analysis showed that *SCN5A* could be readily detected, whereas *SCN9A* was low in hiPSC-CM cells (Figure 7(a)). We next determined if the absence of *SCN9A* in hiPSC-CMs was due to our inability to detect *SCN9A* message. In the next series of experiments, we transfected both *SCN5A* and *SCN9A* into TSA201 at a 1 : 1 molar ratio and probed for message. Results show that both *SCN5A* and *SCN9A* message could be found in transfected TSA201 cells (Figure 7(b)) indicating that *SCN9A* can be readily detected under our experimental conditions and that the message of *SCN9A* is very low or absent in hiPSC-CMs. Similarly, we probed for PXDNL and its homolog, PXDN, in both WT and MMRL1126 hiPSC-CM. Results show that PXDN message was detected in hiPSC-CM, but that PXDNL expression was very low in hiPSC-CMs, as similarly reported [29].

**Figure 8:**
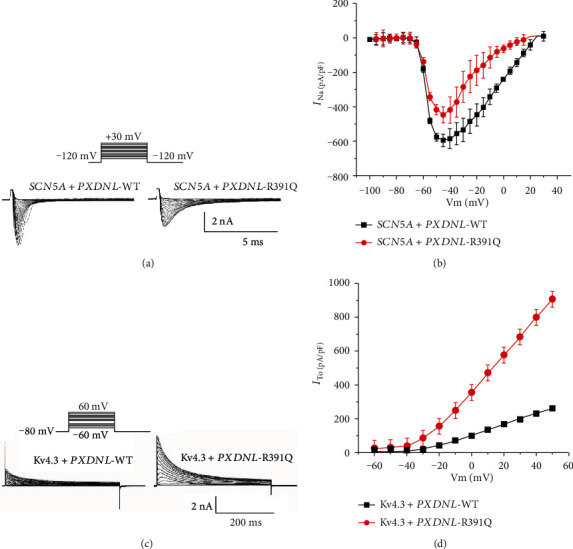
(a) Representative *I*_Na_ recordings from a WT and R391Q mutant in PXDNL cotransfected with SCN5A. I–V relation for WT and R391Q mutant in PXDNL cotransfected with SCN5A. The I-V relation was generated using same voltage clamp protocol described in Figure 5. A small reduction in *I*_Na_ density was noted with R391Q mutation. Representative *I*_to_ recordings from a WT and R391Q (a) mutant in PXDNL cotransfected with KCND3. Current recordings were obtained at test potentials between −60 and 50 mV from a holding potential was −80 mV. (c) I–V relation for WT and R391Q showing a significant difference in I_to_ magnitude at potentials above -20 mV.

Since PXDNL expression is very low in hiPSC-CMs, it seemed likely that any potential interaction of the protein with ion channels may not be observed in hiPSC-CM due to the low levels of PXDNL. In the first set of experiments, we transiently transfected either WT or R391Q PXDNL together with SCN5A. Similar to data shown in Figure 5, *I*_Na_ was activated by a series of depolarizing pulses from -80 to +45 mV in 5 mV increments. The current-voltage relation showed *I*_Na_ was significantly reduced for R391Q compared to WT (Figure 8(b), *p* < 0.05). In the next set of experiments, we transiently transfected either WT or R391Q PXDNL together with KCND3 and recorded *I*_to_. *I*_to_ was activated by a series of depolarizing pulses from -60 to +50 mV in 10 mV increments (Figure 8(c)). The current-voltage relation showed that peak *I*_to_ was significantly greater in R391Q mutant at potentials above -20 mV (Figure 8(d), *p* < 0.05).

**Figure 9:**
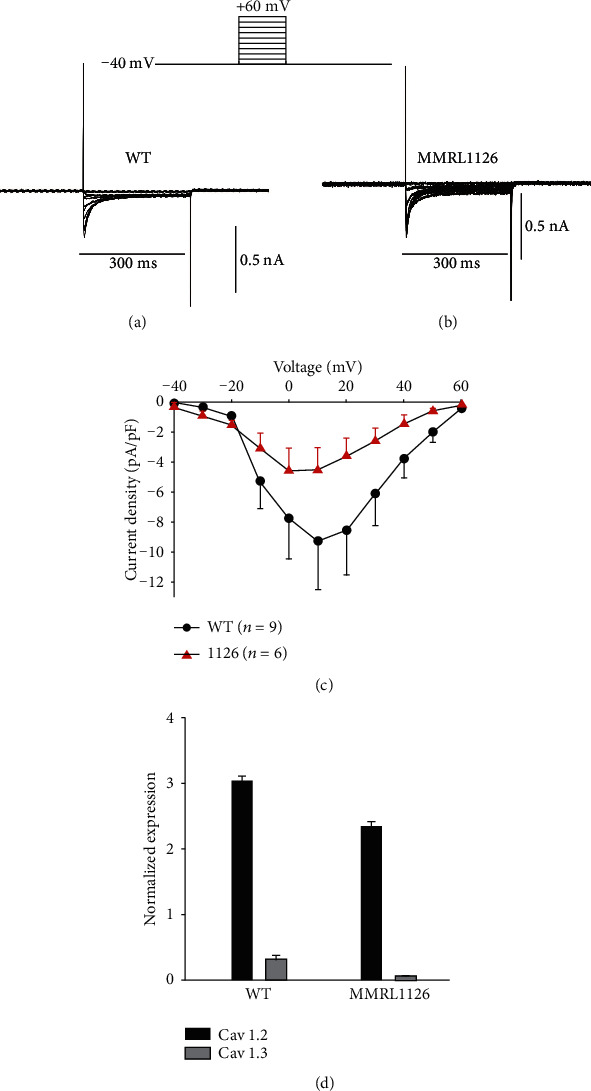
Representative traces showing *I*_Ca_ recorded from (a) WT and (b) MMRL1126. Ca^2+^ currents were recorded during a 300 ms step depolarization from -40 to +50 mV in 10 mV increments. (c) Current-voltage relationship for *I*_Ca_. (d) RT-PCR showing a reduction of Cav1.2 and Cav1.3 message in MMRL1126.

The rise phase of the Ca^2+^ transient is governed by a combination of Ca^2+^ influx through Ca^2+^ channels and Ca^2+^ release from intracellular stores. Since the magnitude of the Ca^2+^ transient was smaller in hiPSC-CM derived from MMRL1126, we speculated that the mutation in *FKBP1b* indirectly affected the size of the Ca^2+^ current. In the next series of experiments, *I*_Ca_ was recorded under voltage-clamp conditions. Following a series of 3 prepulses (prepulses not shown) *I*_Ca_ was activated by a series of depolarizing pulses from -40 to +50 mV in 10 mV increments.  Figure 9(a) shows representative *I*_Ca_ traces from a control and patient-derived hiPSC-CM in response to the voltage clamp protocol. The threshold for *I*_Ca_ activated at -30 mV and peaked at +10 mV. The current-voltage relation showed that *I*_Ca_ density was smaller in MMRL1126 hiPSC-CMs compared to control (Figure 9(b)). Finally, RNA expression of Ca^2+^ channel *α*-subunits was examined by RT-PCR analysis. We examined both isoforms of L-type Ca^2+^ channels (Cav1.2 and Cav1.3). Our results show the lower *I*_Ca_ density observed in MMRL1126 was paralleled by lower message of Cav1.2 and Cav1.3 (Figure 9(c)).

## 4. Discussion

In this study, we identified a patient (MMRL 1126) who initially presented with signs of Early Repolarization Syndrome and monomorphic VT when infused with dobutamine. His syncope was clearly related to his sustained monomorphic VT. Following ablation of potential arrhythmic foci, patient subsequently developed right bundle branch block. Ajmaline challenge unmasked a BrS ECG. Genetic analysis of the patient showed the presence of mutations in three genes, *FKBP1b*, *SCN9A*, and *PXDNL*, none of which had previously been shown to be associated with ERS, BrS, or RBBB. All family members underwent genetic testing, and pedigree analysis revealed that the proband inherited all 3 mutations from the parents. Carriers of the single or double mutation, both parents and sister, did not display any overt clinical phenotype. In addition, hiPSC-CMs derived from the unaffected father (MMRL 1239) also did not show any abnormalities in terms of beating frequency and Ca^2+^ transient magnitude. ERS and BrS are often referred to as J-wave syndromes [35] as they exhibit many similarities and both predispose to life-threatening ventricular arrhythmias. Development of these arrhythmias has previously been associated with alterations in *I*_Na_, *I*_Ca_, *I*_K,ATP_, and *I*_to_ due to ion channel mutations [19, 36, 37]. In our study, patch-clamp recordings of *I*_Ca_ in hiPSC-CMs derived from the patient revealed that *I*_Ca_ was reduced compared to WT hiPSC-CMs suggesting that a reduction in this current may contribute to the mixed clinical phenotype.

Surprisingly, the ECG abnormalities manifested only in the individual who carried 3 mutations. It is unclear which gene(s) played the greater role in the development of the phenotype, since all genes identified in the proband have not previously been shown to be associated with any cardiac arrhythmia syndrome disease. The *PXDNL* gene encodes for a peroxidasin-like protein which is expressed in the heart [29]. Interestingly, the PXDNL proteins encoded by primate genomes appear to lack peroxidase activity suggesting no apparent functional role of the PXDNL protein in human heart [29]. In hiPSC myocytes, PXDNL message and protein were expressed at low levels [29], an observation confirmed in our study. In contrast, adult human ventricular sections showed staining mainly at the intercalated discs with some staining at the lateral surface [29] demonstrating that PXDNL protein is expressed at low levels in neonatal tissue but increase with age and maturity. Whether the presence of this protein at the intercalated disc affects cardiac conduction in adult ventricle and contributes to either the BrS or ERS phenotype remains to be determined. It is worth noting that we have screened 403 patients using NextGen sequencing and have discovered 67 individuals with PXDNL variations at a minor allele frequency of 3% or below (75 variations in total as some individuals had multiple variations). The high association of PXDNL variations in patients exhibiting cardiac arrhythmias coupled with the strong expression of the protein in the ventricles (specifically the intercalated discs) suggests a link between this protein and the development of cardiac arrhythmias.

The role of the *SCN9A* in cardiac tissue is unclear but a similar disconnection between expression and functional role of *SCN10A* in cardiac tissue has been described [38–40]. In our study, PCR analysis of SCN9A message was performed in WT hiPSC-CM and revealed that message was very low in hiPSC-CMs (Figure 6). Consistent with our observations, no cardiac abnormalities have been identified in patients with either a gain [32] or complete loss of function of Nav1.7 [31]. To our knowledge, one study has noted the presence of Nav1.7 in hiPSC-CMs [41]. Although previous studies have demonstrated a role for “brain” type Na^+^ channels in adult heart [30, 42, 43], our results suggest that Nav1.7 message is very low in hiPSC-CMs suggesting a minor role of “brain” type Nav1.7 in hiPSC-CMs. Whether Nav1.7 protein is expressed and has a functional role in adult human heart is unclear (for review see Zimmer et al. [44]).

The role of the *FKBP1B* mutation in MMRL1126 is readily apparent in our study. We were able to detect FKBP1b message in hiPSC-CM, and functional analysis of the calcium transient showed dramatic differences in fluorescence intensity and spontaneous beating rate compared to WT hiPSC-CMs. Patch clamp recordings of *I*_Ca_ in hiPSC-CMs derived from MMRL1126 showed about a 50% amplitude reduction in peak Ca^+^ current compared to WT. Previous studies have demonstrated that a reduction in peak *I*_Ca_ secondary to mutation in Cav1.2 channel subunits can contribute to both the BrS [45, 46] or ERS phenotype [47]. It is unclear if the episodes of syncope and palpitations in this patient are related to ERS, BrS, or RBBB. Using hiPSC-CMs from the proband, we demonstrate that the Ca^2+^ transient is altered in spontaneously active hiPSC-CMs. Since we demonstrate that hiPSC-CMs exhibited altered calcium cycling, a similar situation may be occurring in the patient, likely explaining the syncope following dobutamine infusion.

The manifestation of ERS and BrS (J-wave syndromes) is due to an imbalance in depolarizing and/or repolarizing currents in the early parts of the cardiac action potential. A decrease in inward *I*_Na_ or *I*_Ca_ will accentuate the spike-and-dome morphology of the action potential mainly in the epicardium (epi) generating a transmural voltage gradient that leads to the characteristic ECG changes [13, 14]. In our study, the mutation in *FKBP1B* resulted in alterations in intracellular Ca^2+^ cycling as well as reduced *I*_Ca_ in hiPSC-CMs derived from the patient when compared to WT hiPSC-CMs. In addition, the *PXDNL* mutation resulted in a reduced *I*_Na_ when compared with WT *PXDNL*. Together, mutations in *FKBP1b*, *SCN9A*, and *PXDNL* have a multifactorial effect on the reduction of the two currents resulting in the clinical phenotype. The *PXDNL* variant in addition to producing a loss of function of *I*_Na_ caused a significant increase in *I*_to_, which may have added contributions with the reductions in *I*_Na_ and *I*_Ca_.

The modelling of complex arrhythmia syndromes in hiPSC-CMs has been performed but with variable results, likely due to the immaturity of pluripotent cells as well as the methods used in the various laboratories to derive hiPSC-CMs. For example, several studies have attempted to model BrS in patient-derived hiPSC-CMs but with limited success due largely to the immaturity of the transient outward current in hiPSC-CM [48]. Studies have suggested that ion channel analysis in hiPSC myocytes are superior compared to mammalian expression systems due to the presence of auxiliary subunits [49]. However, other studies have reported no differences in the electrophysiological characteristics (assessed by action potential and microelectrode array recordings) between stem cell-derived cardiomyocytes from a Brugada Syndrome patient and those derived from controls [50]. These observations suggest that hiPSC-CMs are not effective in recapitulating complex phenotypes (such as BrS) where factors such as fibrosis, cellular coupling/uncoupling, and epi- to endorepolarization differences are important.

## 5. Conclusions

Many ventricular arrhythmia syndromes have been linked to mutations in *SCN5A* [51, 52], *CACNA1C*, *CACNB2*, and *CACNA2D1* [45–47]. The latter genes form the L-type calcium channel complex and mutations in these genes produced a reduction in the size of *I*_Ca_ resulting in BrS/ERS phenotypes. Our patch clamp studies showed that *I*_Ca_ was also reduced in hiPSC-CM from MMRL1126. Although 3 family members carried either one or two mutations, none of them has developed arrhythmias to date. In contrast, the proband who inherited all 3 mutations developed palpitations, syncope, and displayed ERS and BrS phenotypes. Using hiPSC myocytes derived from the patient, we demonstrate a clear alteration in several physiological parameters such as reduced ion current density and altered excitation contraction coupling when compared to normal hiPSC myocytes. Consistent with our findings in this study, certain arrhythmia syndromes, such as BrS and ERS, are polygenic in nature [53] with contributions from two or more genetic variants. The results of our study highlight the complexed interaction between genetic mutations and phenotypic expression and demonstrate the effectiveness of hiPSC myocytes manufactured from the patient to study the phenotypic changes.

## Figures and Tables

**Table 1:** MMRL1126 87 gene panel results.

# locus	Gene	Exon	Protein	Coding	Sift	Polyphen	dbsnp
chr2:24283733	FKBP1b	3	p.Lys48fs	c.136delC	VUS	VUS	rs200092869
chr7:91714911	AKAP9	41	NEGATIVE				rs1063242
chr2:167128974	SCN9A	17	p.Ser1085Pro	c.3253T>C	Damaging	Possibly damaging	rs12720442
chr8:52366156	PXDNL	14	p.Arg391Gln	c.1172G>A	VUS	VUS	rs79394014
chr2:167262238	SCN7A	25	NEGATIVE				rs80098689
chr2:166900411	SCN1A	11	NEGATIVE				rs121918769

**Table 2:** Calcium transient parameters from hiPSC-CMs.

	Spontaneous cycle length	Number with regular spontaneous cycle length	% with regular spontaneous cycle length	(*F* − *F*_0_)/*F*_0_
Control	1857.8 ± 56.1 ms (*n* = 102)	102/102	100%	3.83 ± 0.29 (*n* = 102)
MMRL 1239	2310.8 ± 136.2 ms (*n* = 70)	70/75	93.3%	3.78 ± 0.24 (*n* = 93)
MMRL 1126	2802.2 ± 186.9 ms (*n* = 59)	59/93	63.4%	2.03 ± 0.29 (*n* = 93)

**Table 3:** Electrophysiological parameters.

	APD50	APD90	*V*_max_	Maximim diastolic membrane potential
Control	168.0 ± 12.5 ms (*n* = 25)	217.4 ± 13.9 ms (*n* = 25)	38.5 ± 2.4 V/s (*n* = 25)	−68.7 ± 1.1 mV (*n* = 25)
MMRL1126	119.2 ± 7.6 ms (*n* = 13)	163.0 ± 9.4 ms (*n* = 13)	17.3 ± 1.6 V/s (*n* = 13)	−57.8 ± 2.1 mV (*n* = 13)

5 monolayers from MMRL1126 exhibited continuous spontaneous activity; 8 monolayers from MMRL1126 exhibited episodic activity.

## Data Availability

The data that support the findings of this study are available on request from the corresponding author at jcordeiro@mmri.edu.
